# Aspirin Induces Mitochondrial Ca^2+^ Remodeling in Tumor Cells via ROS‒Depolarization‒Voltage-Gated Ca^2+^ Entry

**DOI:** 10.3390/ijms21134771

**Published:** 2020-07-05

**Authors:** Itsuho Fujikawa, Takashi Ando, Manami Suzuki-Karasaki, Miki Suzuki-Karasaki, Toyoko Ochiai, Yoshihiro Suzuki-Karasaki

**Affiliations:** 1 Department of Dermatology, Nihon University Hospital, Tokyo 101-830, Japan; ifujikawa079@gmail.com (I.F.); toochiai@jcom.home.ne.jp (T.O.); 2 Plasma ChemiBio Laboratory, Nasushiobara, Tochigi 329-2813, Japan; ksmanami181@gmail.com (M.S.-K.); ksmiki181@gmail.com (M.S.-K.); 3 Department of Orthopedic Surgery, Yamanashi University School of Medicine, Yamanashi 409-3898, Japan; andou@yamanashi.ac.jp

**Keywords:** aspirin, salicylate, melanoma, apoptosis, depolarization, mitochondria, voltage-gated Ca^2+^ entry, l-type Ca^2+^ channel

## Abstract

Aspirin (acetylsalicylic acid) and its metabolite salicylate, have an anti-melanoma effect by evoking mitochondrial dysfunction through poorly understood mechanisms. Depolarization of the plasma membrane potential leads to voltage-gated Ca^2+^ entry (VGCE) and caspase-3 activation. In the present study, we investigated the role of depolarization and VGCE in aspirin’s anti-melanoma effect. Aspirin and to a lesser extent, salicylate (≥2.5 mM) induced a rapid (within seconds) depolarization, while they caused comparable levels of depolarization with a lag of 2~4 h. Reactive oxygen species (ROS) generation also occurred in the two-time points, and antioxidants abolished the early ROS generation and depolarization. At the same concentrations, the two drugs induced apoptotic and necrotic cell death in a caspase-independent manner, and antioxidants and Ca^2+^ channel blockers prevented cell death. Besides ROS generation, reduced mitochondrial Ca^2+^ (Ca^2+^_m_) and mitochondrial membrane potential preceded cell death. Moreover, the cells expressed the Ca_v_1.2 isoform of l-type Ca^2+^ channel, and knockdown of Ca_v_1.2 abolished the decrease in Ca^2+^_m_. Our findings suggest that aspirin and salicylate induce Ca^2+^_m_ remodeling, mitochondrial dysfunction, and cell death via ROS-dependent depolarization and VGCE activation.

## 1. Introduction

Melanoma is one of the most highly malignant skin cancers, and patients with advanced or metastatic melanoma have a poor prognosis. Recently, the BRAF-MEK inhibitor has served as the standard approach for BRAFV600E/K-mutant advanced melanoma. The BRAFi/MEKi combination therapy trial (COMBI-d) indicates that the combination of dabrafenib and trametinib achieved durable survival in patients with BRAFV600E/K-mutant stage III C unresectable or stage IV metastatic melanoma and supported long-term first-line use of this therapy [1]. However, the efficacy of the BRAFi/MEKi combination therapy was considerably compromised by acquired resistance. Anti-programmed death-1checkpoint-inhibitor therapy causes significant improvements in clinical outcomes in patients with advanced melanoma [2]. Nivolumab has received approval as adjuvant therapy for patients undergoing resection of stage IIIB, IIIC, or IV melanoma. Specifically, the adjuvant use of nivolumab resulted in significantly extended recurrence-free survival [3]. However, such therapy can disturb the immune system homeostasis in patients, resulting in autologous attacks that damage non-malignant organs and tissues, including the lung, intestine, liver, kidney, hormone gland, and skin. These problems can sometimes become severe or life-threatening. Therefore, other novel approaches for melanoma treatment are urgently required.

Aspirin (acetylsalicylic acid) is a well-known nonsteroidal anti-inflammatory drug (NSAID) that acts as an effective antipyretic and analgesic drug. The anti-inflammatory actions of NSAIDs are primarily mediated by their inhibition of prostaglandin synthesis [4]. Aspirin and other NSAIDs have emerged as cancer-preventive drugs. An increasing body of clinical and epidemiological evidence indicates that prolonged use of these drugs can reduce the risks of cancers in gastrointestinal organs as well as those in the breast, prostate, lung, and skin [5]. Numerous studies have indicated that NSAIDs elicit their biological effects independently of cyclooxygenase (COX) inhibition (for a review see, [6]). Specifically, they affect a variety of intracellular signaling pathways, including the mitogen-activated protein kinase cascade, ribosome S6 kinase, signal transducer and activator of transcription 1, and transforming growth factor‒β pathways. They also have modulatory effects on various processes, such as cell cycle progression and activities of nuclear receptor family members, including peroxisome proliferator-activated receptor-γ. These biological effects have roles in tumor growth inhibition and cancer chemoprevention, but it remains unclear whether the effects are direct or indirect [7]. Cell growth inhibition and cell death may contribute to chemoprevention because aspirin was shown to exhibit anti-melanoma effects in vitro and in vivo. It induced apoptosis and necrosis in melanoma cells in COX‒dependent or ‒independent manners [8,9,10,11,12]. Aspirin has very low toxicity and has long been used in clinical practice because of its safety. Therefore, elucidation of the mechanisms underlying its anti-melanoma effect may help in the development of safer cancer treatments involving aspirin.

A decrease in cell volume called the apoptotic volume decrease (AVD) is a hallmark of apoptosis and arises through deregulated movements of ions, mainly monovalent cations likes K^+^ and Na^+^ and disruption of intracellular ion homeostasis [13,14]. AVD was reported to facilitate caspase-3 activation [13]. AVD generation requires ion transport activity across the cell membrane through channels such as Cl^−^ and K^+^ channels and impairment of ion channels or transporters involved in the movements of Na^+^, K^+^, Cl^−^, and Ca^2^^+^ can disrupt intracellular ion homeostasis, leading to depolarization of the plasma membrane potential and apoptosis. Accordingly, depolarization is an early event in the apoptosis induced by diverse agents, including Fas, A23187, rotenone, and arsenic trioxide [15,16,17]. We previously demonstrated that death receptor ligation causes depolarization within 2~4 h and that persistent depolarization by K^+^ loading and inhibition of ATP-sensitive K^+^ channel (K_ATP_) activity augments the anticancer effect of tumor necrosis factor-related apoptosis-inducing ligand (TRAIL) via mitochondrial oxidative stress and endoplasmic reticulum (ER) stress. We also showed the mutual regulation of depolarization and mitochondrial ROS generation through altered oxidative phosphorylation (OXPHOS) [18,19,20]. Furthermore, we recently demonstrated that depolarization is necessary for pro-apoptotic mitochondrial network aberrations [21]. On the other hand, depolarization has anti-apoptotic effects in certain circumstances. Membrane-depolarizing agents, including ouabain, tetraethylammonium, and veratridine, protected Purkinje cells against apoptosis [22]. Thus, depolarization has a dual effect on apoptosis, depending on the cell type involved and apoptotic stimulus applied.

Aspirin and salicylate have been shown to evoke mitochondrial dysfunction. These two drugs induced loss of mitochondrial membrane potential (ΔΨ_m_), decreased ATP production, and increased ROS generation in different cell types [23,24,25]. However, little is known about their effects on depolarization and role in mitochondrial dysfunction. As voltage-gated Ca^2+^ channels (VGCCs) are activated by depolarization to evoke voltage-gated Ca^2^^+^ entry (VGCE), one of the major pathways for extracellular Ca^2^^+^ transport in mammalian cells, VGCE may mediate the cytocidal or cytoprotective effect of depolarization. In this study, we investigated the effect of aspirin and salicylate on tumor cell survival with a particular interest in depolarization and VGCE.

## 2. Results

### 2.1. Aspirin and Salicylate Reduce Tumor Cell Viability in a ROS-Dependent Manner

**Figure 1:**
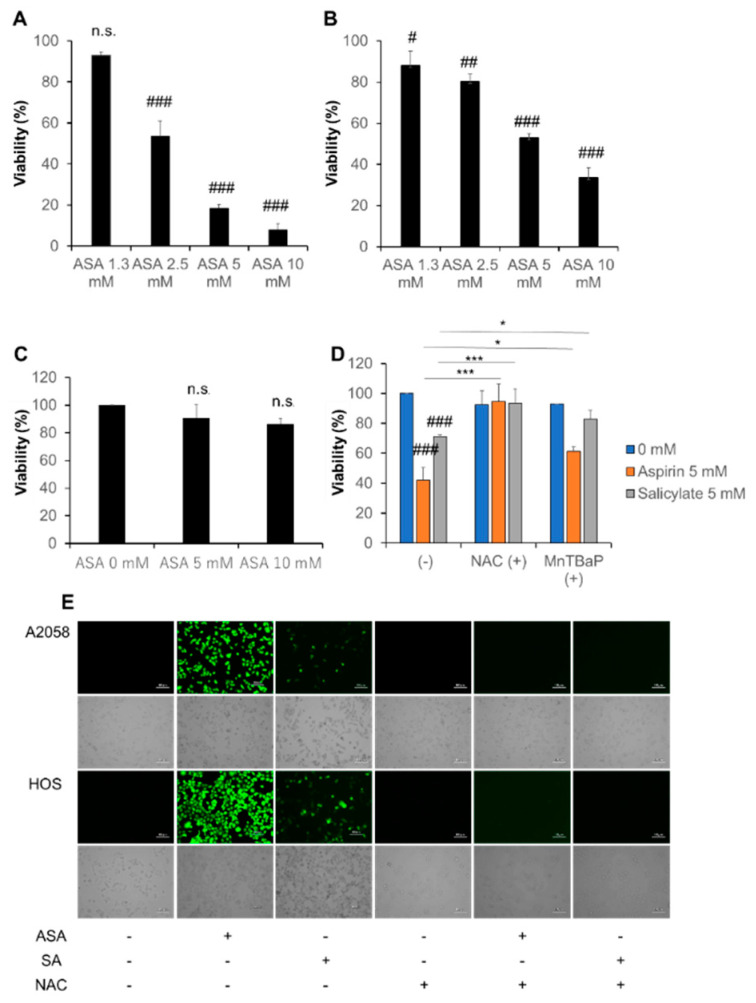
Aspirin and salicylate reduce cell viability in melanoma cells in a reactive oxygen species (ROS)-dependent manner. (**A**) A375, (**B**) A2058, and (**C**) human dermal fibroblasts (HDF) cells in DMEM supplemented with 10% fetal bovine serum (FBS) (FBS/DMEM) were treated with the indicated concentrations of aspirin (ASA) for 72 h at 37 ˚C and analyzed for their viability by the WST-8 assay. Data represent the mean ± SD (*n* = 3). # *p* < 0.05; ## *p* < 0.01; ### *p* < 0.001; n.s., not significant, vs. control. (**D**) A375 cells were treated with aspirin or salicylate in the absence or presence of MnTBaP (30 µM) and NAC (2 mM) for 72 h at 37 °C and analyzed for their viability as described above. Data represent the mean ± SD (*n* = 3). ### *p* < 0.001 vs. control. * *p* < 0.05; *** *p* < 0.001. (**E**) A2058 and HOS cells in FBS/DMEM were cultured in 6-well plates for 24 h and then exposed to aspirin (ASA, 5 mM), salicylate (SA, 5 mM) or NAC alone or in combination for 15 min. Cells were labeled with DCFH-DA FITC antibody and observed with a fluorescence microscope.

WST-8 cell growth assays revealed that aspirin (≥2.5 mM) dose-dependently decreased the viability of A375 and A2058 cells with having a more significant effect in A375 cells (Figure 1A,B). Aspirin also dose-dependently decreased cell viability in HOS and MG63 cells (Figure S1A,B), while it (≤10 mM) had minimal effect on the viability of human dermal fibroblasts (HDF) (Figure 1C). Salicylate had smaller effects in melanoma (Figure 1D) and osteosarcoma cells (data not shown). The sensitivity to aspirin and salicylate varied considerably in different experiments. Usually, both drugs (≥5 mM) showed significant effects in melanoma cell lines (10 mM aspirin caused a maximum of 90% reduction in A375 cells). Moreover, antioxidants prevented these effects. Specifically, superoxide dismutase mimetic Manganese (III) tetrakis (4-benzoic acid)porphyrin chloride (MnTBaP) and *N*-acetylcysteine (NAC) significantly inhibited the effect of aspirin and salicylate (Figure 1D). Moreover, massive ROS production was observed in live A2058 and HOS cells as rapidly as 15 min after the addition of aspirin or salicylate, although aspirin was much more potent than salicylate (Figure 1E). The addition of NAC also abolished these responses. These results indicate that aspirin and salicylate reduce tumor cell viability in a ROS-dependent manner. Because melanoma cell lines have high sensitivity to aspirin and salicylate, we investigated their cytotoxic effects in more detail using them as a model.

### 2.2. Aspirin and Salicylate Induce Apoptotic and Necrotic Cell Death

**Figure 2:**
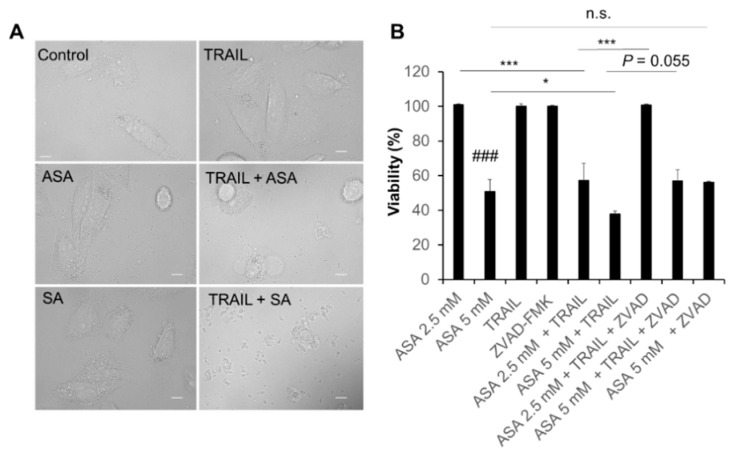
Aspirin and salicylate induce melanoma cell death. (**A**) A2058 cells treated with aspirin (ASA) or salicylate (SA) (5 mM) and TRAIL (100 ng/mL) alone or in combination for 24 h at 37 ˚C were observed under a BZX-710 all-in-one biological microscope and analyzed using the BZ-H3A application software. Scale bars, 10 µm. (**B**) Cells were treated with the indicated concentrations of aspirin (ASA) and TRAIL (100 ng/mL) alone or in combination with the absence or presence of Z-VAD-FMK (10 µM; ZVAD) for 72 h at 37 °C and were analyzed for viability by the WST-8 assay. Data represent the mean ± SD (*n* = 3). ### *p* < 0.001 vs. control. * *p* < 0.05; *** *p* < 0.001; n.s., not significant.

Treatment with aspirin or salicylate (≤5 mM) alone for 24 h had minimal effects on the morphology of A2058 cells (Figure 2A). TRAIL alone also caused minimal changes in cellular morphology. Nevertheless, when aspirin and TRAIL were used together, massive cell expansion, a hallmark of necrotic cell death, was observed. Meanwhile, the combined use of salicylate and TRAIL led to severe cell membrane destruction and cell body shrinkage (Figure 2A). Consistent with these observations, aspirin acted synergistically with TRAIL to decrease viability in the cells. While TRAIL (100 ng/mL) alone minimally reduced cell viability (<10%), it significantly potentiated the effect of aspirin (≥2.5 mM) (Figure 2B). The pan-caspase inhibitor Z-VAD-FMK completely inhibited the sensitization to aspirin (2.5 mM) and tended to reduce the sensitization to aspirin (5 mM), but minimally reduced the cell death caused by aspirin (5 mM) alone (Figure 2B).

To determine the cell death modality, we performed double staining with fluorescein isothiocyanate (FITC)-conjugated annexin V and propidium iodide (PI) after drug treatment. Flow cytometry analyses showed that aspirin and salicylate increased apoptotic (annexin V-positive) and necrotic (annexin V-negative, PI-positive) A375 cells at concentrations that reduced cell viability. Aspirin and salicylate increased apoptotic cells in a dose-dependent manner, while necrotic cells were increased maximally at concentrations of 5 and 2.5 mM, respectively (Figure S2A,B). TRAIL alone modestly increased both cell populations. Consistent with the WST assay results, both aspirin and salicylate synergistically increased apoptotic and necrotic cell death with TRAIL in these cells (Figure S2A,D,E). Either drug (≥10 mM) alone induced a high degree of apoptotic cell death (>80%) (Figure S2B,C).

### 2.3. Aspirin and Salicylate Induce Mitochondrial Dysfunction

We examined the effect of aspirin on mitochondrial depolarization and ROS generation to determine the role of the mitochondrial death pathway. Flow cytometry measurements using JC-1, a mitochondrial-targeting ratiometric dye, showed that aspirin or salicylate (≥2.5 mM) significantly reduced ΔΨ_m_ in a dose-dependent manner and that high concentrations (≥5 mM) of the two drugs completely abolished ΔΨ_m_ (Figure S3A,B). After aspirin treatment, the signal for the ROS probe dihydroethidium increased in a dose-dependent manner. Aspirin (≥2.5 mM) showed this effect with 5- and 10-mM aspirin increasing the signal by 9.3-fold and 6.6-fold, respectively (Figure S3C). Similarly, 5- and 10-mM salicylate increased the signal by 11.4-fold and 6.8-fold, respectively (Figure S3D). These results indicate that aspirin and salicylate induce mitochondrial dysfunction.

### 2.4. Aspirin Rapidly Evokes Depolarization in a ROS-Dependent Manner

**Figure 3:**
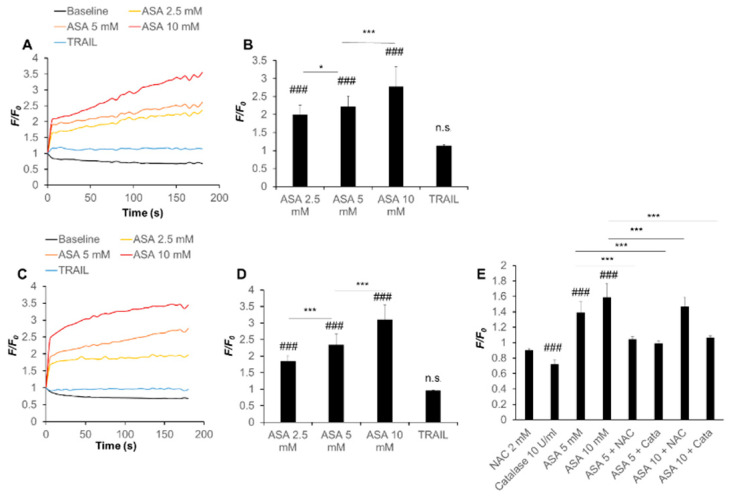
Aspirin evokes rapid and persistent depolarization in a ROS-dependent manner. (**A‒D**) (**A**,**B**) A375 and (**C**,**D**) A2058 cells loaded with DiBAC4(3) were washed, resuspended in HBSS, and treated with the indicated concentrations of aspirin (ASA) or TRAIL (100 ng/mL). (**E**) A375 cells loaded with the same probe were treated with the indicated concentrations of ASA in the absence or presence of NAC and catalase. The cells were measured for their fluorescence for 3 min in a microplate fluorescence reader with excitation and emission at 485 and 538 nm, respectively. The trace with the vehicle alone is considered as a baseline. Data are shown as *F/F_0_*, where *F* and *F_0_* are the sample’s fluorescence at each time point and time zero, respectively. Graphs in (**B**,**D**,**E**) represent the mean ± SD of the average *F/F_0_* (*n* = 3). ### *p* < 0.001; n.s., not significant vs. baseline. * *p* < 0.05; *** *p* < 0.001.

Previously, we have shown that substantial depolarization of the plasma membrane occurs in parallel with mitochondrial ROS production and ΔΨ_m_ collapse during melanoma cell apoptosis and that there is a mutual regulation among them [20]. Therefore, we determined the ability of aspirin to affect the plasma membrane potential. Analyses with the anionic voltage-dependent fluorescent dye DiBAC4(3) revealed that aspirin (≥2.5 mM) evoked robust depolarization in A375 cells in a dose-dependent manner (2.3- to 3.5-fold increase). The effect was observed within seconds and lasted throughout the monitored period (3 min) without repolarization (Figure 3A,B). Similar results were obtained in A2058 cells (1.8- to 3.3-fold increase) (Figure 3C,D). The effect of aspirin (5 mM) was comparable while that of aspirin (10 mM) was significantly higher than that of K^+^ loading, used as a positive control. Salicylate also caused depolarization with similar kinetics to a lesser extent. Salicylate (10 mM) increased depolarization only by ~1.2-fold in both cell types (Figure S4A,B). Meanwhile, TRAIL (100 ng/mL) caused minimal changes in the plasma membrane potential during the monitored period (Figure 3A‒D). Flow cytometric analyses showed the occurrence of a late depolarization. Specifically, even at 4 h after stimulation, the DiBAC4(3) signal was increased by 2.9‒ fold and 3.2‒fold in aspirin (5 mM)- and (10 mM)-treated cells, and by 3.3‒ and 3.2‒fold in salicylate- (5 mM) and (10 mM)-treated cells, respectively. At the same time point, the signal was 1.6-fold higher in TRAIL-treated cells compared with control cells. Unlike late depolarization, early depolarization was not always significant, because massive repolarization occurred under certain circumstances. We speculated that specific cellular factors regulated the rapid response. Because ROS regulate the late depolarization caused by TRAIL [20], we hypothesized that ROS also controlled the effect of aspirin/salicylate and examined the ability of several antioxidants to affect this response. As expected, catalase and NAC significantly blocked the early depolarization caused by aspirin (≥5 mM) (Figure 3E). These results indicate that aspirin rapidly evokes depolarization in a ROS-dependent manner.

### 2.5. Ca^2+^ Regulates the Anti-Melanoma Effect of Aspirin

**Figure 4:**
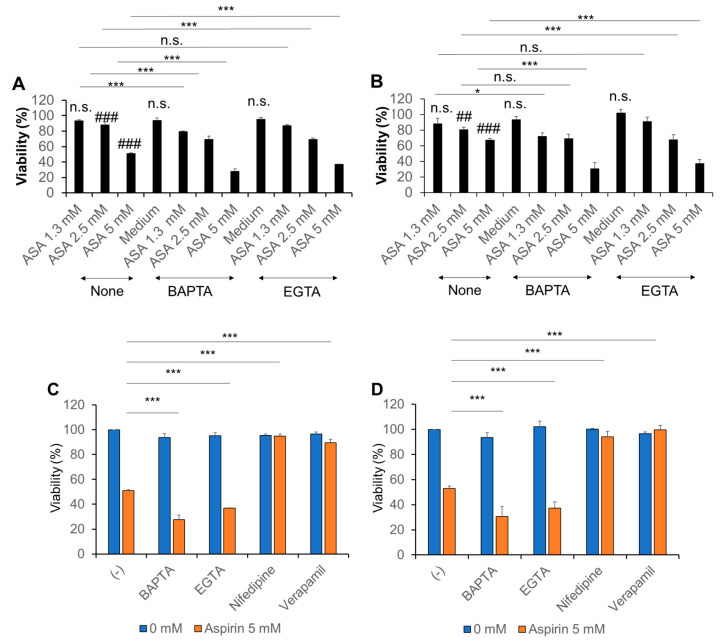
Ca^2+^ regulates the anti-melanoma effect of aspirin. (**A**,**B**) (**A**) A375 and (**B**) A2058 cells were treated with the indicated concentrations of aspirin (ASA) in the absence or presence of BAPTA-AM (30 µM) and EGTA (0.2 mM) for 72 h and analyzed for viability by the WST-8 assay. Data represent the mean ± SD (*n* = 3). ### *p* < 0.001; n.s., not significant vs. control. * *p* < 0.05; *** *p* < 0.001. (**C**,**D**) (**C**) A375 and (**D**) A2058 cells were treated with aspirin in the absence or presence of BAPTA-AM (30 µM), EGTA (0.2 mM), and nifedipine or verapamil (1 µM) for 72 h and analyzed for viability as described above. Data represent the mean ± SD (*n* = 3). *** *p* < 0.001.

To gain insight into the role of Ca^2+^ in the anti-melanoma effect of aspirin, we examined the effect of Ca^2+^ removal. For this purpose, cells were treated with aspirin in the absence or presence of BAPTA-AM and EGTA as chelators of intracellular and extracellular Ca^2+^, respectively. Both compounds alone minimally affected cell viability but significantly augmented the decrease in viability of A375 and A2058 cells induced by aspirin (Figure 4A,B). BAPTA tended to be more potent than EGTA. In contrast, we found that Ca^2+^ channel blockers targeting l-type Ca^2+^ channels (LTCCs) such as nifedipine and verapamil, completely prevented cell death induced by aspirin. The Ca^2+^ channel blockers specifically inhibited aspirin cytotoxicity, because Ca^2+^ chelators augmented the cytotoxicity under the same conditions (Figure 4C,D). These results suggest that aspirin can evoke pro-survival and pro-death Ca^2+^ signals and that LTCCs may mediate the pro-death Ca^2+^ signal.

### 2.6. Aspirin Modulates the Intracellular Ca^2+^ Dynamics 

**Figure 5:**
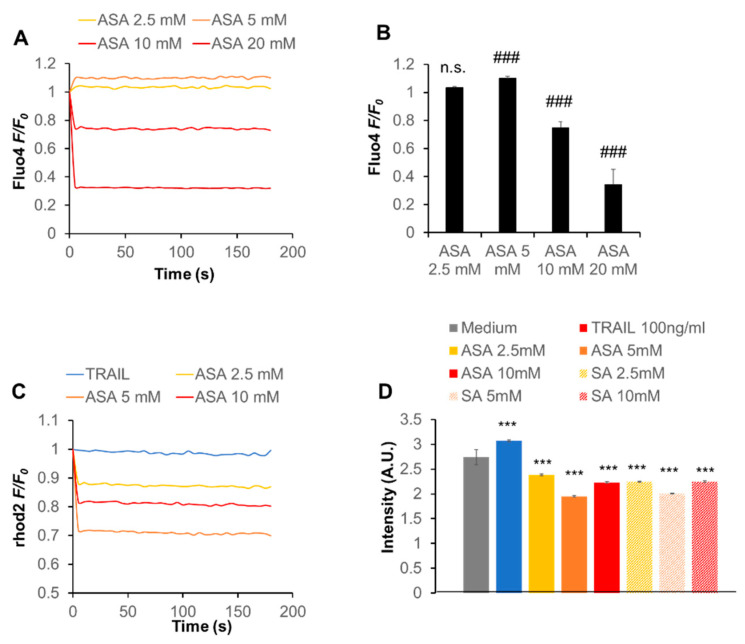
Aspirin modulates the intracellular Ca^2+^ dynamics in melanoma cells. (**A**,**B**) A2058 cells were loaded with Fluo4-AM, treated with the indicated concentrations of aspirin (ASA), and immediately measured for their fluorescence for 3 min with excitation and emission at 485 and 538 nm, respectively, in a microplate reader. Data are shown as *F/F_0_*, where *F* and *F_0_* are the sample’s fluorescence and control, respectively, and represent the mean ± SD (*n* = 3). *** *p* <0.001; n.s., not significant vs. control. (**C**,**D**) Cells were loaded with dihydrorhod 2-AM, treated with the indicated concentrations of ASA or salicylate (SA) and TRAIL (100 ng/mL), and measured for their fluorescence for 3 min with excitation and emission at 542 and 592 nm, respectively. In (**C**), data are shown as *F/F_0,_* where *F* and *F_0_* are the fluorescence of the sample and control, respectively. In (**D**), data are shown as fluorescence intensity (arbitrary units, [A.U.]) and represent mean ± SD (*n* = 3). *** *p* < 0.001 vs. unstimulated control.

To investigate the functional link between Ca^2+^ and the anti-melanoma effect in more detail, we examined whether aspirin affected Ca^2+^ dynamics. Ca^2+^ measurements revealed that aspirin had a dual effect on the cytosolic Ca^2+^ concentration ([Ca^2+^]_c_), depending on the concentration: aspirin at ≤ 5 mM increased [Ca^2+^]_c_ while aspirin at higher concentrations decreased it in a dose-dependent manner (Figure 5A,B). Meanwhile, aspirin decreased the mitochondrial Ca^2+^ (Ca^2+^_m_) concentration ([Ca^2+^]_m_) with a maximal effect at 5 mM (Figure 5C). Similar effects were observed with salicylate (Figure 5D). The decrease in Ca^2+^_m_ uptake was specific for these two drugs because TRAIL minimally decreased [Ca^2+^]_m_ or rather increased it in the cells (Figure 5C,D). These results indicate that aspirin modulates the intracellular Ca^2+^ dynamics, including Ca^2+^_m_.

### 2.7. Ca_v_1.2 Downregulation Inhibits the Effect of Aspirin on Ca^2+^_m_

**Figure 6:**
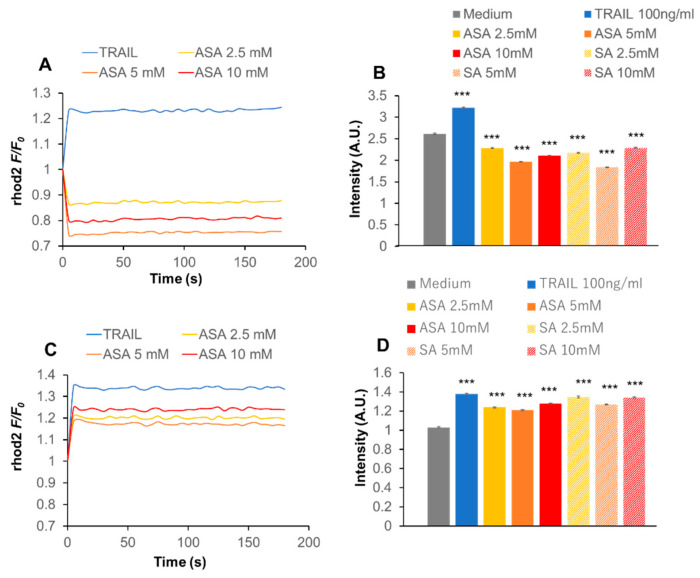
Ca_v_1.2 downregulation modulates the effect of aspirin on Ca^2+^_m_. (**A‒D**) (**A**,**B**) Control (scrambled control siRNA-transfected) and (**C**,**D**) Ca_v_1.2 knockdown A375 cells were treated with the indicated concentrations of aspirin (ASA), salicylate (SA), and TRAIL and [Ca^2+^]_m_ was measured as described in the legend of Figure 2. In (**A**) and (**C**), data are shown as *F/F_0_*_,_ where *F* and *F_0_* are the fluorescence of the sample and control, respectively. In (**B**) and (**D**), data are shown as fluorescence intensity (arbitrary units, [A.U.]) and represent the mean ± SD (*n* = 3). *** *p* < 0.001 vs. unstimulated control.

We analyzed LTCC’s isotype expression in the cells because VGCE was implicated in the anti-melanoma effect of aspirin in A375 and A2058 cells (Figure 4C,D). We focused on the expression of the Ca_v_1.2 and Ca_v_1.3 isoforms, because they are commonly expressed in various human cancer cells [26,27,28]. Semiquantitative RT-PCR analysis using primers specific for Ca_v_1.2 and Ca_v_1.3 revealed that the cells expressed the two transcripts at varying degrees. A2058 cells expressed both Ca_v_1.2 and Ca_v_1.3 transcripts, while A375 cells expressed Ca_v_1.2 substantially but Ca_v_1.3 only modestly (Figure S5A,B). Next, we attempted to analyze the function of the isoforms. Cells were transfected with small interfering RNAs (siRNAs) targeting Ca_v_1.2 or Ca_v_1.3 to downregulate the expression of each isoform with minimally affecting the expression of the other isoform. Eventually, we succeeded in downregulating the expression of Ca_v_1.2 with minimal reduction in the expression of Ca_v_1.3 in A375 cells (Figure S5C). Subsequently, Ca_v_1.2 knockdown and control (scrambled control siRNA-transfected) cells were treated with aspirin, and [Ca^2+^]_m_ was measured. We observed a significant (38%) reduction in the basal [Ca^2+^]_m_ level in Ca_v_1.2 knockdown cells compared to control cells. As observed in A2058 cells (Figure 5D), aspirin and salicylate decreased [Ca^2+^]_m_ with a maximal effect at 5 mM in control cells (Figure 6A,B). On the contrary, the two drugs significantly increased [Ca^2+^]_m_ in Ca_v_1.2 knockdown cells (Figure 6C,D), indicating that the Ca_v_1.2 knockdown inhibited the effect. Meanwhile, the Ca_v_1.2 knockdown affected the effect of TRAIL only modestly.

## 3. Discussion

The data presented in this study show that aspirin has anti-melanoma and TRAIL adjuvant activities. Consistent with previous reports [8,9,10,11,12], aspirin induced cell death in TRAIL-resistant melanoma cell lines. Aspirin also reduced cell viability in human osteosarcoma cells (Figure S1). Meanwhile, aspirin had much a smaller cytotoxic effect in human fibroblasts, indicating that aspirin preferentially acts on tumor cells. Aspirin alone increased both apoptotic and necrotic cell death (Figure S2), which was unaffected by the pan-caspase inhibitor Z-VAD-FMK. In contrast, this compound completely abolished synergistic cell death induction by TRAIL and aspirin (Figure 2B). Also, no significant cleavages of caspase−8, −9, −3, and PARP were seen after aspirin treatment at a range of concentrations (2.5, 5, and 10 mM) and time points (6, 12, and 24 h) (data not shown). These findings suggest that aspirin can induce different cell death modalities alone or with TRAIL. Aspirin also induced ROS generation in live cells virtually at once (Figure 1), which was followed by robust ΔΨ_m_ dissipation and another ROS generation with a lag of 2~4 h (Figure S3), and antioxidants prevented the effect and cell death (Figure 1D,E). Collectively, mitochondrial dysfunction and ROS may play a vital role in the anti-melanoma effect. 

Aspirin can elicit various biological effects in a COX-dependent or -independent manner [4,6]. Aspirin’s ability to acetylate the enzyme through its acetyl group primarily mediates the COX-dependent effect [4]. Accordingly, salicylate lacking an acetyl group in its structure fails in COX inactivation through acetylation of the enzyme. Nevertheless, salicylate had an anti-melanoma effect. It also induced the same levels of ΔΨ_m_ dissipation and the second ROS generation (Figure S3). Strikingly, however, salicylate made a much smaller level of the first ROS generation (Figure 1E). Notably, these two phases of ROS generation could be attributed to different oxidant species. They were measured with different oxidant probes DCFH-DA and DHE, and DCFH-DA specifically reacts with peroxides mainly H_2_O_2_ but not superoxide, while DHE reacts preferentially with superoxide. If it was the case, aspirin and salicylate have different abilities to stimulate hydrogen peroxide. Meanwhile, they seemed to be equipotent in dissipating ΔΨ_m_ dissipation and the late ROS generation. Notably, we have demonstrated that late superoxide generation mainly results from OXPHOS inhibition and is critical for TRAIL adjuvant activity [18,19,20]. Meanwhile, early ROS production might result from NAD(P)H oxidase activation. Thus, aspirin and salicylate might have substantially similar effects on OXPHOS in a COX-independent manner. Further investigations to test this view are underway.

At concentrations that induced cell death, aspirin evoked strong and robust depolarization, and antioxidants blocked these effects (Figure 3). These findings indicate that the depolarization is mediated by ROS and involved in the anti-melanoma effect of aspirin. Notably, the depolarization occurred at early and late stages during cell death; the early depolarization occurred within seconds, while the late depolarization occurred after several hours. Meanwhile, TRAIL caused late depolarization, but not early depolarization (Figure 3). Moreover, only late depolarization was significant in some experiments. Thus, these two phases of depolarization may occur through different pathways and ROS. Because the kinetics of the two phases of depolarization and ROS are comparable, early and late depolarization likely results from early and late ROS production, respectively. Notably, different types of depolarization occur by distinct mechanisms during apoptosis within a particular cell type. For example, both Na^+^ and Cl^−^ fluxes contribute to arsenic trioxide-induced depolarization but not anti-Fas-induced depolarization [17]. Also, several redox-sensitive ion pumps and channels, including Na^+^-K^+^-ATPase and K_ATP_, have critical roles in proapoptotic depolarization [16,18]. Thus, these ion fluxes, pumps, and channels might participate in the two types of depolarization. Further investigations are necessary to clarify the mechanisms of depolarization after aspirin treatment.

Ca^2^^+^ has a dual effect on cell survival depending on the magnitude, timing, duration, and spacing of the Ca^2^^+^ surge generated. Short and synchronized Ca^2^^+^ waves are necessary for energy production, cell function, and cell survival [29]. On the contrary, excessive and persistent increases in the intracellular Ca^2^^+^ are significant causes of mitochondrial dysfunction, integrity disruption, and cell death by triggering increased permeability of the inner mitochondrial membrane [30,31,32,33]. Store-operated Ca^2^^+^ entry (SOCE) is the principal mechanism for physiological intracellular Ca^2^^+^ rises in a variety of cell types [34]. SOCE is mediated by store-operated channels (SOCs) that are activated by Ca^2^^+^ depletion in the ER. The depletion induces translocation of stromal interaction molecule 1 (STIM1) to ER/plasma membrane junctional regions, where it activates ORAI1 channels on the membrane and induces Ca^2^^+^ influx. SOCE has also emerged as critical machinery for Ca^2^^+^ influx in cancer cells and contributes to various malignant phenotypes [35,36]. Moreover, a mutual regulation between SOCE and Ca^2^^+^_m_ uptake was recently demonstrated. Specifically, Ca^2^^+^ released from the ER was transported to the mitochondrial matrix via the voltage-dependent anion channel–mitochondrial Ca^2^^+^ uniporter pathway, thereby promoting SOCE [35,36]. Meanwhile, SOCE was shown to be necessary for active Ca^2^^+^_m_ uptake [37]. Thus, SOCE has emerged as a critical pro-survival Ca^2^^+^ pathway that could be exploited as a target for cancer treatment [35]. Notably, we observed that the removal of Ca^2^^+^ augmented the anti-melanoma effect of aspirin (Figure 4). The findings were similar to those obtained with two different apoptosis inducers, TRAIL, and the organosulfur compound diallyl trisulfide (DATS) [38,39]. Ca^2^^+^ removal affected cell death only when cells were exposed to these pro-death stimuli, suggesting that specific Ca^2^^+^ signals may be activated to protect cells from stress. Collectively, it is possible to speculate that SOCE is activated to provide a pro-survival Ca^2^^+^ signal in cells upon exposure to these pro-apoptotic agents. In support of this idea, Núñez and colleagues showed that salicylate inhibited SOCE, thereby reducing Ca^2+^_m_ uptake and cell proliferation in Jurkat leukemia and colon cancer cells [23].

Another important pathway for extracellular Ca^2^^+^ entry is VGCE, which occurs through various types of VGCCs. Because depolarization activates different types of VGCCs depending on its magnitude and duration, they may play a role in mediating the biological effect of aspirin. Consistent with this view, Ca^2^^+^ channel blockers such as nifedipine and verapamil, inhibited the anti-melanoma effect (Figure 4), similar to the case for DATS [39]. Because LTCCs are the primary targets for these compounds, we further investigated their role. Consistent with a previous report by Das and colleagues [28], we found that the melanoma cell lines expressed Ca_v_1.2 and Ca_v_1.3 transcripts. Moreover, we succeeded in downregulating Ca_v_1.2 expression with a minimal effect on Ca_v_1.3 expression (Figure S5). Although the downregulation was incomplete (maximum of 70% reduction, *n* = 3), more severe conditions employing prolonged incubation periods and higher siRNA concentrations caused non-selective effects. Ca_v_1.2 knockdown significantly reduced the ambient Ca^2+^_m_ uptake, indicating that these Ca^2^^+^ channels participate in the basal Ca^2+^_m_ uptake. Moreover, the knockdown abolished the decrease in Ca^2+^_m_ uptake induced by aspirin (Figure 6). Strikingly, aspirin increased Ca^2+^_m_ uptake in the absence of Ca_v_1.2 expression, indicating that other Ca^2^^+^ transport pathways, including Ca_v_1.3, may contribute to the effect. SOCE is also a likely candidate for an aspirin target in the modulation of [Ca^2+^]_m_ because depolarization reduces the negative charge of the cell membrane, as the driving force for SOCE. Moreover, previous studies demonstrated the coordinated control of STIM1-ORAI1 and Ca_v_1.2 [40,41]. Thus, there is an intriguing possibility that Ca_v_1.2 plays a role in Ca^2+^_m_ uptake via a specific interaction with SOCE. Another candidate for an aspirin target in the regulation of [Ca^2+^]_m_ is mitochondrial Na^+^/Ca^2+^ exchanger (mNCLX) because we previously showed that the mNCLX pathway regulated [Ca^2+^]_m_ and apoptosis in melanoma cells [42]. Moreover, mNCLX was shown to regulate [Ca^2+^]_m_ and excitotoxicity in cooperation with VGCCs in neurons [43]. Further studies for clarification of the involvement of mNCLX are ongoing.

**Figure 7:**
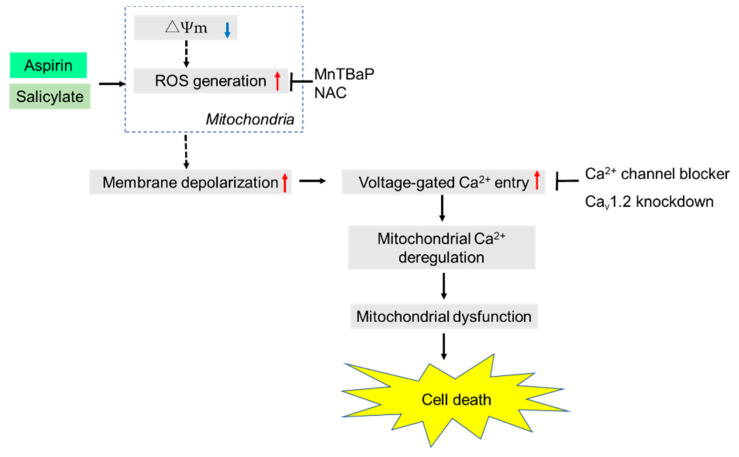
A schematic summary of the present study. Both aspirin and salicylate induce rapid and persistent depolarization, and ROS mediate the effect. The ROS may be primarily generated by the electron transport chain through loss of ΔΨ_m_, as we previously showed with electron transport chain inhibitors, rotenone, antimycin A, and FCCP [20]. In turn, the depolarization without repolarization leads to excessive activation of VGCCs, including Ca_v_1.2, thereby leading to Ca^2+^_m_ deregulation, mitochondrial dysfunction, and cell death.

In conclusion, we have demonstrated that aspirin and salicylate can evoke depolarization and VGCE through Ca_v_1.2 LTCCs, thereby disturbing Ca^2+^_m_ dynamics and leading to mitochondrial dysfunction and cell death in melanoma cells (Figure 7). To the best of our knowledge, this is the first study to demonstrate that aspirin can modulate Ca^2+^_m_ dynamics via depolarization in malignant cells. However, the present study has several experimental limitations. In Western blotting of the pore-forming α_1C_ subunit, we mainly detected multiple bands of ≤ 100 kDa, but not the much larger bands of 180~220 kDa corresponding to the full-length α_1C_ (data not shown). The simplest explanation for this phenomenon is the proteolysis of α_1C_ during the sample preparation process, despite the presence of a standard protease cocktail, but the reason is currently unknown. Extensive studies attempting to address these issues are underway.

## 4. Materials and Methods 

### 4.1. Materials

All chemicals were purchased from Sigma Aldrich (St. Louis, MO, USA) unless otherwise specified. Soluble recombinant human TRAIL was obtained from Enzo Life Sciences (San Diego, CA, USA). The pan-caspase inhibitor Z-VAD-FMK was purchased from Merck Millipore (Darmstadt, Germany). All insoluble reagents were dissolved in dimethylsulfoxide (DMSO) and diluted with high glucose-containing DMEM supplemented with 10% fetal bovine serum (FBS) or Hank’s balanced salt solution (HBSS, pH 7.4; Nissui Pharmaceutical Co., Ltd., Tokyo, Japan) (final DMSO concentration, <0.1%) before use.

### 4.2. Cell Culture

Cells were cultured in 10% FBS/DMEM supplemented with 100 U/mL penicillin and 100 μg streptomycin (Pen-Strep, Thermo Fisher Scientific Japan, Tokyo, Japan) in a 95% air/5% CO_2_ humidified atmosphere. The human melanoma cell line A375 (cell number CRL-1619) was obtained from the American Type Culture Collection (Manassas, VA, USA). The human melanoma cell line A2058 (cell number IFO 50276) and the human osteosarcoma cell line MG63 (IFO50108) were purchased from the Japanese Collection of Research Bioresources Cell Bank of National Institutes of Biomedical Innovation, Health, and Nutrition (Osaka, Japan). The human osteosarcoma cell line HOS (cell number RCB0992) was obtained from Riken BioResource Center (Tsukuba, Japan). Cells were harvested by incubation with 0.25% trypsin-EGTA (Thermo Fisher Scientific, Waltham, MA, USA) for 5 min at 37 °C.

### 4.3. Cell Viability Assay

Cell viability was measured in triplicates by the WST-8 assay using Cell Counting Reagent SF (Nacalai Tesque Inc., Kyoto, Japan) as previously described [42] with modifications. This method is a colorimetric assay based on the formation of a water-soluble formazan product. Briefly, cells seeded in 96-well plates (Corning Incorporated, Corning, NY, USA) at a density of 8 × 10^3^ cells /well were cultured with the agents to be tested for 72 h at 37 °C, followed by addition of 10 μL of cell counting reagent SF and further incubation for 2 h. The absorbances were measured at 450 nm using an ARVO MX microplate reader (PerkinElmer Japan Co., Ltd., Yokohama, Japan).

### 4.4. Measurements of ROS Generation in Live Cells

A2058 and HOS cells were cultured in 6-well plates for 24 h and then exposed to ASA (5 mM), SA (5 mM), or NAC (2 mM) alone or in combination for 15 min. Cells were labeled with DCFH-DA FITC antibody using the reactive oxygen species (ROS) detection assay kit (BioVision, Inc., Milpitas, CA, USA) according to the manufacturer’s instructions. Images were taken in triplicates with fluorescence microscopy FLUOVIEW FV10i (Olympus, Tokyo, Japan) with excitation and emission at 495 and 529 nm, respectively.

### 4.5. Cell Death Assay

Cell death was quantitatively in duplicates assessed by double-staining with FITC-conjugated annexin V and PI as previously described [18]. Briefly, cells plated in 24-well plates (2 × 10^5^ cells /well) were treated with the agents to be tested for 20 h and stained with FITC-conjugated annexin V and PI using a commercially available kit (Annexin V FITC Apoptosis Detection Kit I: BD Biosciences, San Jose, CA, USA). The stained cells (10,000 cells) were analyzed using the FL-1 and FL-2 channels of a FACSCalibur flow cytometer (BD Biosciences) using the CellQuest software (BD Biosciences). Four cellular subpopulations were evaluated: viable cells (annexin V-negative, PI-negative); early apoptotic cells (annexin V-positive, PI-negative); late apoptotic cells (annexin V-positive, PI-positive); and necrotic/membrane-damaged cells (annexin V-negative, PI-positive).

### 4.6. Intracellular Ca^2+^ Measurements

Changes in Ca^2+^_c_ and Ca^2+^_m_ levels were measured in triplicates using Fluo4-AM and rhod2-AM (Dojindo Kumamoto, Japan), respectively, as previously described [38]. To improve the mitochondrial localization of rhod 2-AM, it was reduced to the colorless, nonfluorescent dihydrorhod 2-AM by sodium borohydride treatment, according to the manufacturer’s protocol. Cells were loaded with 4 μM Fluo 4-AM or dihydrorhod 2-AM for 40 min at 37 °C, washed with HBSS, and resuspended at 1 × 10^6^/mL in 96-well plates. The cells were manually treated with the agents to be tested, and measured for their fluorescence in a microplate reader (Fluoroskan Ascent, ThermoFisher Scientific) with excitation and emission at 485 and 538 nm (for Fluo 4-AM), and 542 and 592 nm (for rhod 2-AM), respectively.

### 4.7. Measurement of Depolarization

Depolarization of the plasma membrane potential was measured in triplicates using the anionic bis-oxonol voltage-sensitive dye DiBAC4(3) as previously described [18] with minor modifications. Briefly, cells suspended in HBSS at 1 × 10^6^ cells/mL were incubated with 5 µM DiBAC4(3) (Dojindo Laboratories) for 40 min at 37 °C for dye loading. The cells were then washed, resuspended in HBSS, and measured for their fluorescence for 3 min using a microplate reader (Fluoroskan Ascent; Thermo Fisher Scientific Japan) with excitation and emission at 485 and 538 nm, respectively.

### 4.8. Gene Expression Analyses

For semiquantitative reverse transcriptase-polymerase chain reaction (RT-PCR) analyses, total RNA was isolated using RNA-Bee (Tel Test Inc., Friendswood, TX, USA) and reverse-transcribed to cDNAs with a First-Strand cDNA Synthesis Kit (GE Healthcare Life Sciences, Pittsburgh, PA, USA) using 1 μg of total RNA as a template. The resulting cDNAs were amplified by PCR using a Taq PCR Master Mix Kit (Qiagen, Hilden, Germany) according to the manufacturer’s protocols. The PCR amplifications were performed for 35 cycles comprising 1 min at 94 °C for denaturation, 1 min at 57 °C for annealing, and 1 min at 72 °C for extension. The following primers were used: human Cav1.2 (antisense), 5′-CTCGGACTCTGGGGCACACTTCTT-3′ and (sense), 5′-ACTCCCGCATCT CCATCACCTTCTTC-3′ Cav1.3 (sense), 5′-ACGAGCAGTCCAAGATGTTCAAT-3′; and (antisense), 5′-TCAGAGTTCCCAGGTGTAGCAG-3′. All primers were obtained from Thermo Fisher Scientific. The transcripts were separated by agarose gel electrophoresis and visualized by ethidium bromide staining under ultraviolet light. Quantitative real-time PCR was performed using the previously described primers [28] with modifications. Briefly, total RNA was isolated by an RNAeasy^®^ kit (Qiagen) and reverse-transcribed to cDNAs with an iScript^tm^ cDNA Synthesis Kit (Bio-Rad Laboratories, Hercules, CA, USA). Quantitative PCR was performed using an SYBR^®^Premix ExTaq™ System (Takara Bio Inc., Kusatsu, Japan).

### 4.9. Gene Silencing by RNA Interference

For downregulation of Ca_v_1.2 expression, cells plated in 6-well plates at 2.5 × 10^5^/well were transfected with 5 nM siRNA targeting human Ca_v_1.2 (S2284; Thermo Fisher Scientific) or scrambled control siRNA (Santa Cruz Biotechnology, Santa Cruz, CA, USA) using a Lipofectamine^®^ RNA/Max Kit (Thermo Fisher Scientific) according to the manufacturer’s instructions and cultured for 48 h at 37 °C in a 5% CO_2_-containing atmosphere. Downregulation of the Ca_v_1.2 transcript was assessed by quantitative PCR, as described above.

### 4.10. Statistical Analysis

Data were presented as the mean ± standard deviation (SD), and analyzed by one-way analysis of variance followed by a Tukey post hoc test using add-in software for Excel 2016 for Windows (SSRI, Tokyo, Japan). Values of *p* < 0.05 was considered to be statistically significant.

## Supplementary Materials

Supplementary materials can be found at https://www.mdpi.com/1422-0067/21/13/4771/s1.
