# Supplementary material for: Aspirin Induces Mitochondrial Ca2+ Remodeling in Tumor Cells via ROS‒Depolarization‒Voltage-Gated Ca2+ Entry
Source: Int J Mol Sci. 2020 Jul 5;21(13):4771. doi: 10.3390/ijms21134771 (PMC7370041; doi:10.3390/ijms21134771)
Supplement: Supplementary file 1 [file ijms-21-04771-s001.pdf]

Figure S1

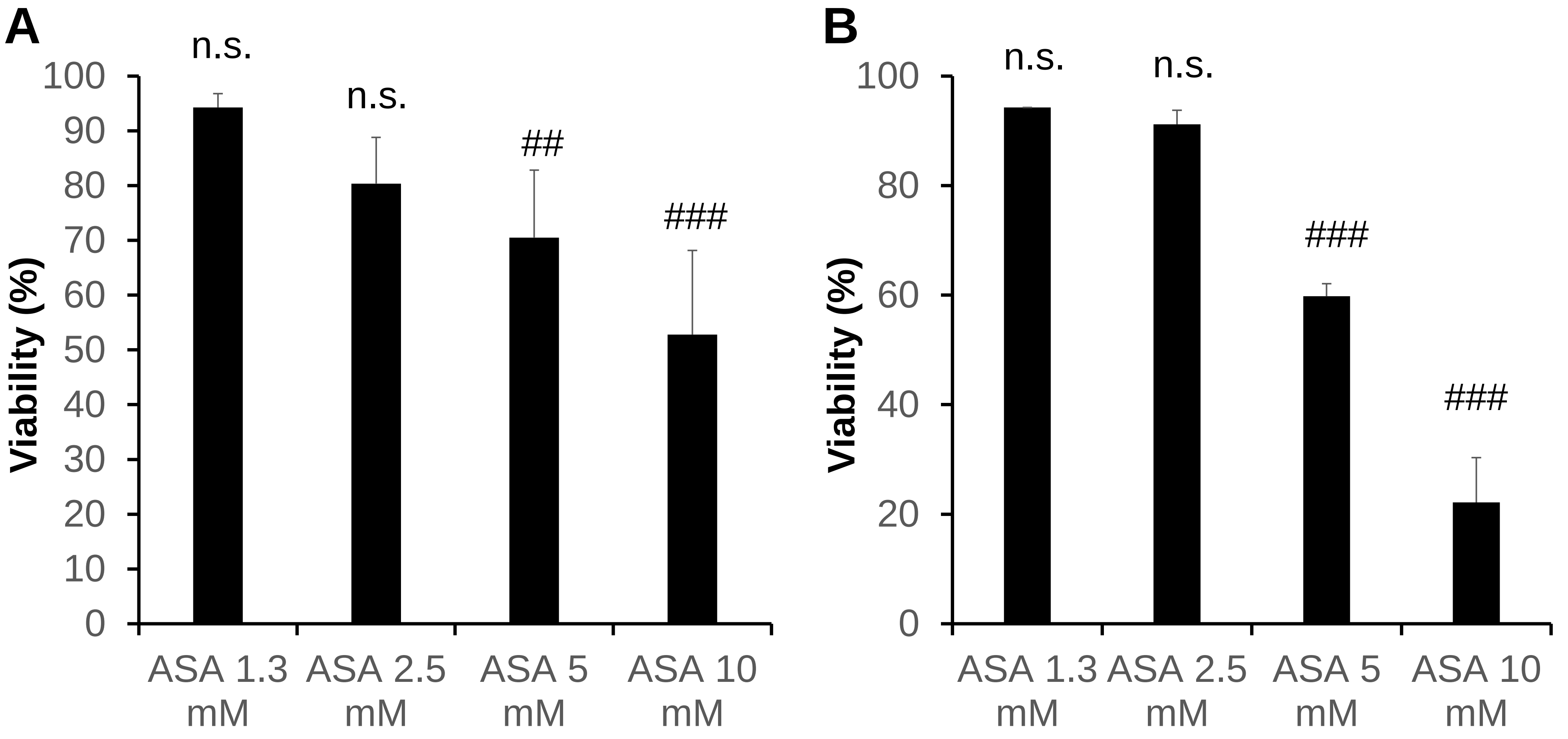

Figure S1. (A) HOS and **(B)** MG63 cells in DMEM supplemented with 10% FBS were treated with aspirin (ASA) at the indicated concentrations for 72 h at 37 ˚C and analyzed for their viability by the WST-8 assay. Data represent the mean ± SD (n = 3). ## *p* < 0.01; ###*p* < 0.001; n.s., not significant, vs. control (ASA 0 mM).

Figure S2

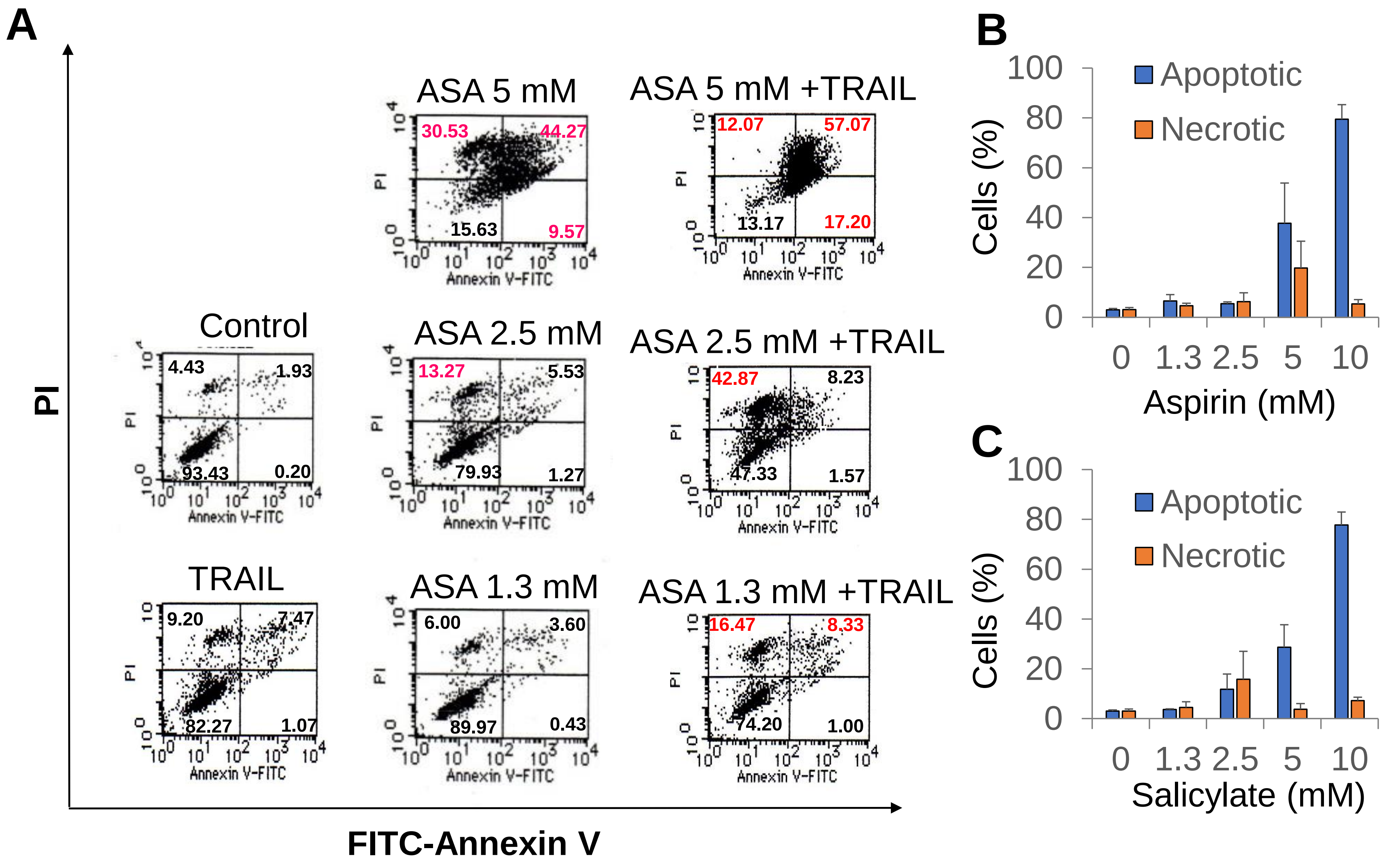

Figure S2. A375 cells treated with the indicated concentrations of aspirin (ASA) or salicylate, and TRAIL (100 ng/mL) alone or in combination for 72 h at 37 ˚C. Then, the cells were stained with FITC-conjugated annexin V and PI. The green fluorescence (annexin V) and red fluorescence (PI) were measured using the FL-1 and FL-2 channels, respectively, of a flow cytometer and analyzed by the CellQuest software. Four subpopulations were evaluated: live cells (annexin V-negative, PI-negative); early apoptotic cells (annexin V-positive, PI-negative); late apoptotic cells (annexin V-positive, PI-positive); and necrotic/cell membrane damaged cells (annexin V-negative, PI-positive). Annexin V-positive cells are considered as apoptotic cells. **(A)** Representative histograms. **(B, C)** Percentages of apoptotic and necrotic cells after treatment with the indicated concentrations of **(B)** aspirin or **(C)** salicylate, respectively. Data represent the mean ± SD (n = 3).

Figure S3

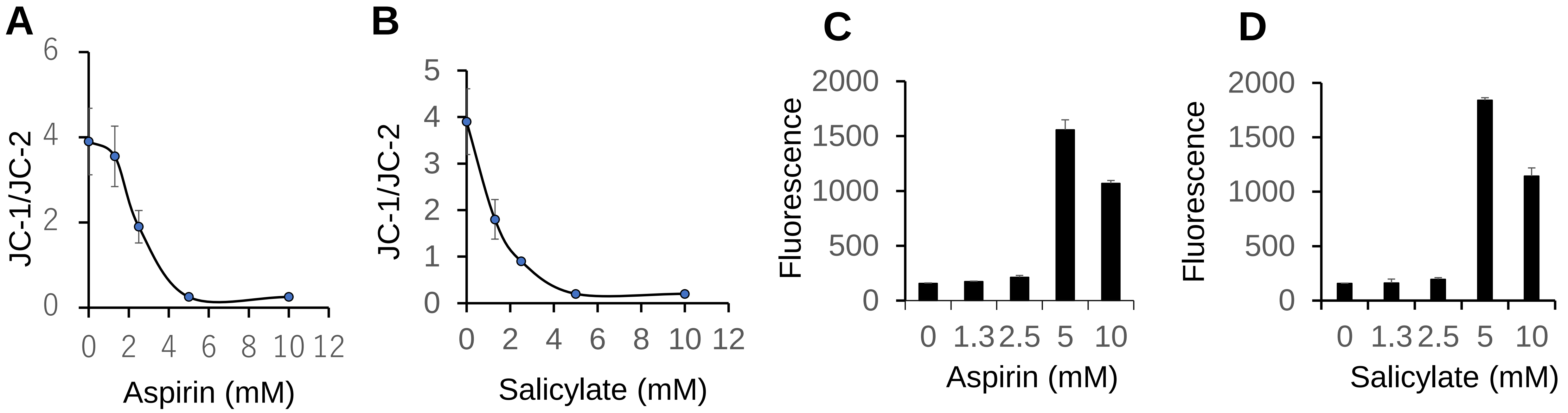

Figure S3. Aspirin and salicylate induce mitochondrial dysfunction. **(A, B)** A375 cells were loaded with JC-1 for 15 min, washed, and treated with the indicated concentrations of **(A)** aspirin or **(B)** salicylate for 4 h. The green fluorescence (monomeric JC-1) and red fluorescence (J-aggregates) were measured using the FL-1 and FL-2 channels, respectively, of a flow cytometer and analyzed by the CellQuest software. **(C, D)** Cells were loaded with dihydroethidium and treated with **(C)** aspirin or **(D)** salicylate for 4 h. The red fluorescence was measured using the FL-2 channel of the flow cytometer. Data represent the mean $\pm$ SE (n = 3).

Figure S4

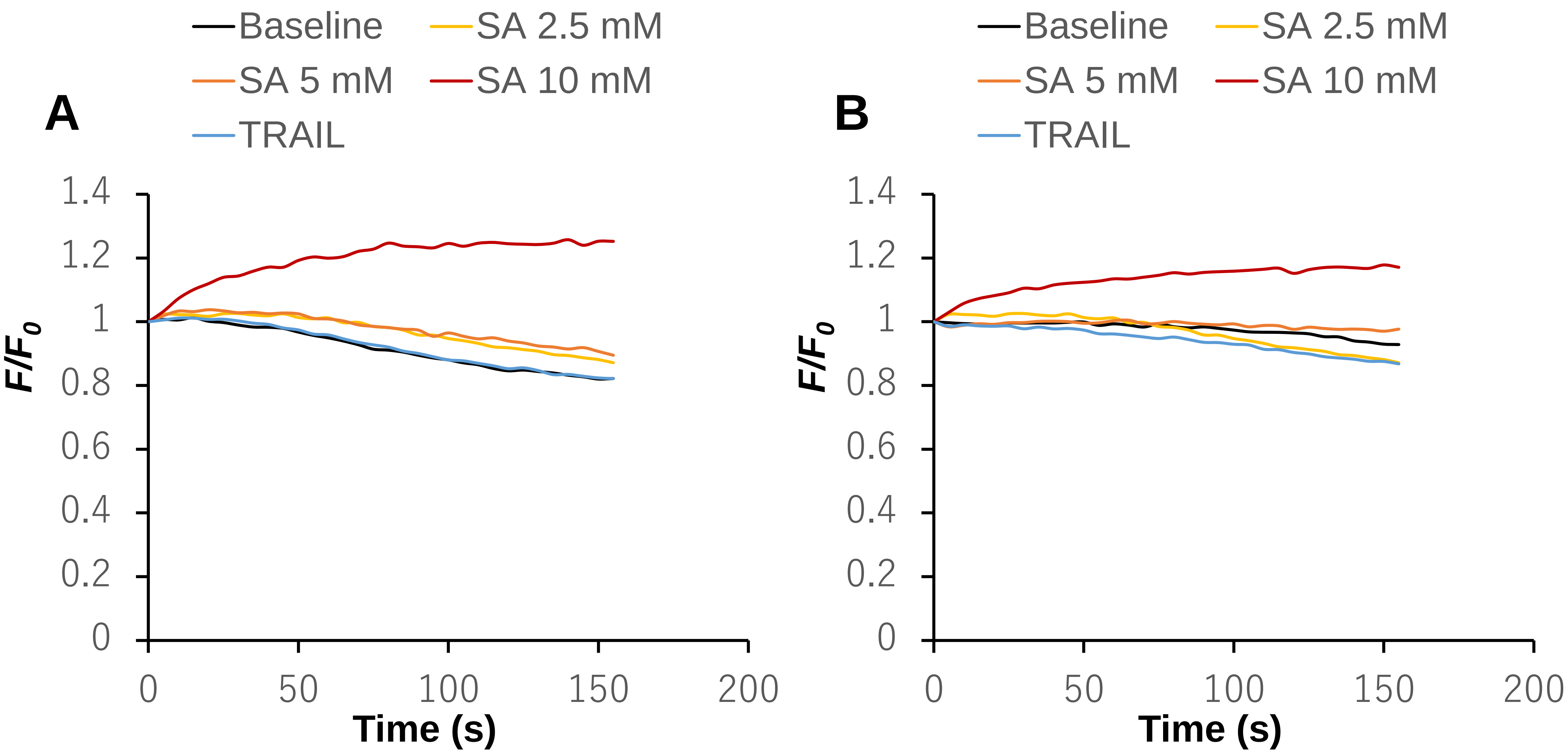

Figure S4. (A) A375 and **(B)** A2058 cells were loaded with DiBAC4(3), washed, and resuspended in HBSS. After addition of the indicated concentrations of salicylate (SA) or TRAIL (100 ng/mL), the cells were measured for their fluorescence using a microplate reader with excitation and emission at 485 and 538 nm, respectively. The trace with the vehicle alone is considered as a baseline. Data are shown as F_0/F, where F and F_0 represent the fluorescence at each time point and zero time, respectively. Data represent the mean ± SD (n = 3).

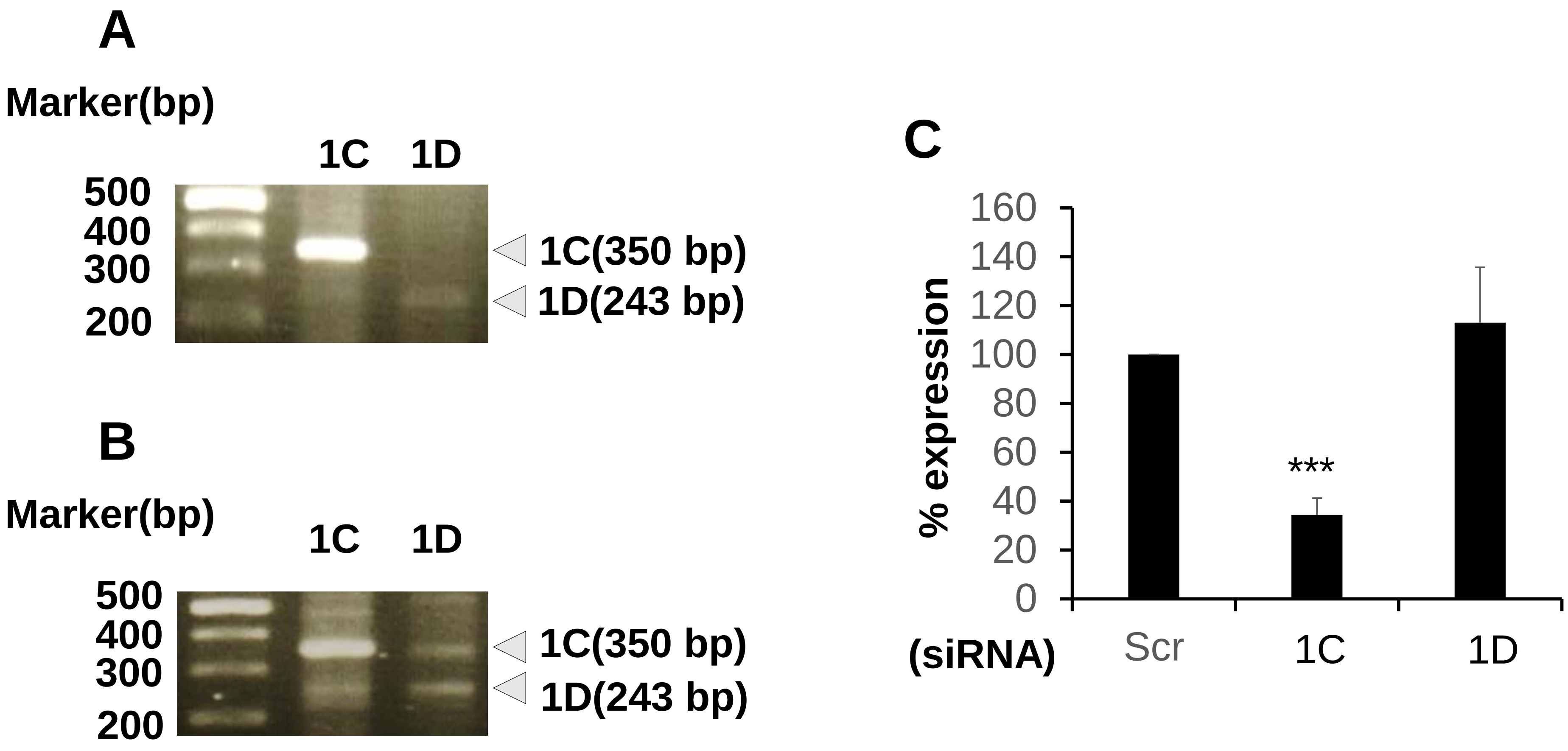

Figure S5. Expression of Ca$_v$1.2 and Ca$_v$1.3 in melanoma cells. **(A, B)** Total RNAs were isolated from **(A)** A375 and **(B)** A2058 cells and subjected to cDNA synthesis. The resulting cDNAs were amplified by RT-PCR. The Ca$_v$1.2 (1C) and Ca$_v$1.3 (1D) transcripts were detected using specific primers. GAPDH was evaluated as a loading control. **(C)** A375 cells were transfected with a siRNA targeting human Ca$_v$1.2 (1C) or Ca$_v$1.3 (1D) or a scrambled control siRNA (Scr) using a Lipofectamine^R RNA/Max Kit and cultured for 48 h. The expression levels of Ca$_v$1.2 and Ca$_v$1.3 transcripts were assessed by quantitative PCR. Data represent the mean $\pm$ SD (n =3). ***p <0.001 vs. Scr.
